# State Medicine, Public Health, Hygiene and Bacteriology

**Published:** 1895-05

**Authors:** Ernest Wende

**Affiliations:** Health Commissioner of the City of Buffalo, N. Y.


### Progress in Medical Science.

### STATE MEDICINE, PUBLIC HEALTH, HYGIENE AND BACTERIOLOGY.

Conducted by ERNEST WENDE, M. D.,
Health Commissioner of the City of Buffalo, N. Y.

### Work in the Bacteriological Bureau, Department of Health, Buffalo, N. Y.

WILLIAM G. BISSELL, M. D., Bacteriologist.

On account of the many inquiries which have been received at the Buffalo Bacteriological Laboratory regarding the bacterial examination of suspected cases of diphtheria, it has been thought desirable to give a condensed account of the facts regarding the work, together with the methods employed and the reliability of the same.

#### DIPHTHERIA.

In order to do this satisfactorily, it will be necessary to state in brief the more important investigations made which demonstrates that diphtheria is due to the organism described by Löffler and is primarily a local disease.

In 1883, a microörganism, peculiar and striking in appearance, was found by Klebs[1] to be of constant occurrence in the false membrane of persons dying of true epidemic diphtheria. In 1884, Löffler[2] published the result of extensive investigations on the subject. He found the microörganism described by Klebs (a bacillus) in nearly all cases of throat inflammation which had been previously diagnosticated as diphtheria. He succeeded in isolating the organism in pure culture and found that when inoculated upon the abraded mucous membrane of susceptible animals, it produced a false membrane, frequently followed by death of the animal. He also demonstrated that by injecting subcutaneously the proper amount of a bouillon culture, death could be produced in guineapigs with characteristic diphtheritic lesions. Löffler failed to find the organism in every case examined by him, and that is now fully explained by the fact that in certain varieties of pseudo-membranous inflammation, especially like those occurring in scarlet fever, the organism was not found, such inflammation being erroneously considered at that time to be true diphtheria. In 1888, Roux and Yersin[3] published the first portion of a series of thorough investigation. They found the organism in all cases of characteristic diphtheria and found that the properties of the organism, as described by Löffler, were entirely correct. They demonstrated that when the organism was inoculated on the healthy mucous membrane of the trachea of rabbits, no result followed, but if the mucous membrane was abraded, a typical diphtheritic lesion was the result. Roux and Yersin produced characteristic diphtheritic paralysis in animals, getting this result principally in pigeons and rabbits, which had been inoculated with cultures of the organism and did not succumb to a too rapid intoxication, the paralytic symptoms in the case of a pigeon developing twenty-one days after the apparent recovery of the animal. Quoting directly from the author, he states: "The occurrence of these paralyses, followed by the introduction of the bacilli of Klebs-Löffler, completes the resemblance of the experimental disease to the natural malady and establishes with a certainty the specific rule of this bacillus."

Among other more recent investigators are Welch and Flexner[4], Prudden,[5] Babes[6] and others, and it is now generally conceded to be a fact by bacteriologists and men of experience that this organism is the etiological factor in the production of diphtheria.

Some claim that it does not appear to be the only factor, and that seems true, for, as a rule, it requires some abnormal condition—a reduction in the vital capacity of the human organism—for the invasion of the disease and this in a way applies to all infectious maladies.

Under the name "diphtheria," therefore, should be included all inflammations of the mucous membrane, due to the bacillus of Klebs-Löffler. An acute hyperemia, caused by this organism, is as truly diphtheritic as an inflammation with a pseudo-membrane or exudate. As to how much reliance can be placed on the bacteriologic examination in the diagnosis of diphtheria, I quote directly from a recent report[7] of the original work on this subject under the direction of Dr. Hermann M. Biggs in the Bacteriological Department of the City of New York:

After a year's trial and thorough investigation the following conclusions were arrived at: the examination by a competent bacteriologist of the bacterial growth in a blood serum tube which has been properly inoculated and kept for fourteen hours at the body temperature, can be thoroughly relied on in cases where there is visible membrane in the throat, if the culture is made during the period in which the membrane is forming and no antiseptic, especially no mercurial solution, has lately been applied.

In cases where the disease is confined to the larynx or bronchi and where, therefore, there is no visible exudate against which the swab can be rubbed, surprisingly accurate results can be obtained from the examination of cultures, but in a certain proportion of cases no diphtheria bacilli will be found in the first culture and yet will be abundantly present in later ones, the bacilli having probably been coughed up more freely as the disease progressed. We believe, therefore, that absolute reliance for a diagnosis cannot be placed on a negative result in a single culture from the pharynx in purely laryngeal cases.

In nasal diphtheria a negative result may be obtained from a culture made from the throat and yet the bacilli be found in cultures from the nose.

In making a diagnosis from a culture, it is essential to know the duration of the disease in the case from which it was made, because, although bacilli may remain present and alive in some throats for many weeks, it is, nevertheless, important to remember they may vanish early and suddenly and that, therefore, the cultures cannot be certainly relied on after the membrane begins to disappear.

The use of antiseptics shortly before making the inoculation of a culture tube may render the culture useless for diagnosis. It has been found in a few instances that a culture made from a case of diphtheria, shortly after a thorough irrigation with a 1-4000 solution of bi-chloride of mercury, gave no diphtheria bacilli, though one made just before and one made some time later gave them abundantly. It is a curious fact that under such circumstances a vigorous growth of other organisms may take place.

The above conclusions are true only when the inoculations have been properly made and, in judging cultures received from physicians in general, the greatest care must be taken. Some cultures are made carelessly and some evidently without taking the pains even to read the instructions or to glance at the condition of the coagulated serum in the tube. If, therefore, when no diphtheria bacilli are found, the bacterial growth is scanty, the media dry or contaminated, or the inoculation in any way faulty, the case must be referred back for another culture.

The second culture in these cases not infrequently contains the bacilli when the first did not.

The absence of bacilli in a culture proves the case to be one of false diphtheria only when it has been possible to make it under the proper conditions.

The Department of Health in the City of Buffalo will issue in the near future, to the physicians of the city, the following circular :

Department of Health,
Buffalo, N. Y.

*Circular of Information Concerning the Use of Bacterial Cultures for the Diagnosis of Diphtheria.*

Recent bacterial investigations have shown that a considerable proportion of the cases of pseudo-membranous and exudative inflammations of the throat and upper air-passages, commonly considered as diphtheria, and having the anatomical appearances found in diphtheria, are not true diphtheria. These cases may be called pseudo, or false diphtheria.

It has also been shown that a considerable number of cases which are apparently false diphtheria prove, on bacterial examination, to be true diphtheria. While in true diphtheria the mortality is very high and the danger of transmission to others is great, in false diphtheria the mortality is low and the danger of infection slight. The differential diagnosis between the true and false diphtheria can be made, in the greater majority of cases, by bacteriological examination within twenty-four hours, while without this the differentiation is difficult or impossible.

The Health Department is now prepared to make use of bacterial cultures for diagnosis in all cases of suspected diphtheria occurring in the city, and desires that in every case the physicians should themselves make the inoculations. They should be made in every suspicious case at the earliest possible moment, for during convalescence the specific organisms often disappear from the throat, and the full benefit of a positive diagnosis is not obtained, unless it is made early in the disease.

The inoculations are made by gently rubbing a cotton swab against the throat, and then drawing it over the surface of the culture medium. The physician can obtain, free of cost, a culture tube and swab, and the simple directions necessary for their use, at any one of the addresses given on the "pocket card."

After the inoculation the tubes in the box are to be returned at once to the station from which they were obtained, it being necessary to have them there before 3 P. M., or sent directly to the Health Department by 6 P. M.

The diagnosis will be ready by noon the following day. The attending physician can obtain this immediately by telephoning to the department after that time, and when this is not done, he will be notified by mail. Cases which prove to be false diphtheria will not be visited by the Health Department inspectors. Cases, on the other hand, which prove to be true diphtheria, will be subjected to the usual rules and regulations covering contagious diseases.

Experiments have led the department to adopt the rule that no person who has suffered from diphtheria shall be considered free from contagion until it has been shown by bacteriological examination, made after the disappearance of the membrane from the throat, that the throat secretions no longer contain the diphtheria bacilli, and that until such examinations have shown such absence all cases should remain isolated and under observation. Disinfection of the premises, therefore, should not be performed until examination has shown the absence of the organisms.

It has also been found that, occasionally, when culture tubes are inoculated immediately after irrigation of the throat with antiseptic solutions, the cultures do not show any Löffler bacilli, although subsequent examinations may demonstrate their presence. This observation should be noted in making inoculations.

When the bacteriological diagnosis does not harmonize with the clinical facts and the history, as shown by antecedent or subsequent cases of diphtheria, and where there are any defects or reasons for complaint regarding the service in any respect, physicians are earnestly requested to report these promptly to the Health Commissioner. Knowledge of defects in the service can only reach the department through such reports, and the service can only thus be improved and perfected.

Physicians are requested to read carefully the directions accompanying the culture sets, and to follow exactly the instructions given. Thus uniformity in method and accuracy in results will be insured.

ERNEST WENDE, M. D., *Health Commissioner.*

Accompanying this circular will be the referred to "pocket card," which reads as follows:

DEPARTMENT OF HEALTH, BUREAU OF BACTERIOLOGY,
MUNICIPAL BUILDING, DELAWARE AVENUE,
BUFFALO, N. Y.

Directions for inoculating culture tubes with the exudate in cases of suspected diphtheria:

The patient should be placed in a good light, and, if a child, properly held. The swab is removed from its tube, and, while the tongue is depressed, it is passed into the pharynx (if possible, without touching the tongue, and is rubbed gently, but firmly, against any visible membrane on the tonsils or in the pharynx, and then, without laying the swab down, it is immediately inserted in the blood serum tube, and the portion which has been previously in contact with the exudate is rubbed a number of times back and forth over the whole surface of the serum. This should be thoroughly but gently done, so as not to break the surface of the serum. The swab is replaced in its tube, and both tubes, their cotton plugs having been inserted, are returned to the box and sent to the collecting station. The blank forms of report which accompany each outfit should be completely filled out and forwarded to the station with the tubes.

Where there is no visible membrane (it may be present in the nose or pharynx) the swab should be thoroughly rubbed over the mucous membrane of the pharynx and tonsils, and in nasal cases, when possible, a culture should also be made from the nose. In little children, care should be taken not to use the swab when the throat contains food or vomited matter, as then the bacterial examination is rendered more difficult. Under no conditions should any attempt be made to collect the material shortly after the application of disinfectants (especially solutions of corrosive sublimate) to the throat. If any of these instructions have not been carried out, the fact should be carefully noted on the record blank.

List of stations where culture sets may be obtained free of cost: Department of Health, second floor municipal building, Delaware avenue; first precinct station house, corner Franklin and West Seneca streets; second precinct station house, Seneca street, east of Louisiana street; third precinct station house, Pearl street, near Chippewa; fourth precinct station house, Sycamore street, corner of Ash; fifth precinct station house, corner of Emily street and Delavan avenue; sixth precinct station house, Main street, south of Ferry (Cold Springs); seventh precinct station house, Louisiana street, near Elk; eighth precinct station house, 484 William street; ninth precinct station house, Seneca street, corner of Babcock; tenth precinct station house, Niagara street, near Jersey; eleventh precinct station house, Broadway, corner Bailey avenue; twelfth precinct station house, Genesee street, near Parade house; thirteenth precinct station house, corner Austin street and Joslyn place.

Return culture outfit to the station from which it was received before 3 P. M., or to the Department of Health by 6 P. M. A report will be sent by mail the following morning, or can be obtained by telephoning the Department of Health after 12 o'clock noon.

Depending on the results obtained from the examination of the cultures submitted for diagnostic purposes, one of the following forms, which is printed on the back of a postal card, is filled out and mailed to the physician before 12 o'clock noon :

DEPARTMENT OF HEALTH,<br>BACTERIOLOGICAL LABORATORY.

BUFFALO, N. Y., ...... 189..

Dear Dr. ........ The examination of the cultures made by inoculating the tubes with the exudation from the throat of .......... on ...... shows the presence of the diphtheria bacilli.

The case is, therefore, one of true diphtheria.

ERNEST WENDE, M. D., *Health Commissioner.*

............ *Bacteriologist.*

DEPARTMENT OF HEALTH,<br>BACTERIOLOGICAL LABORATORY.

BUFFALO, N. Y., ......... 189..

Dear Dr ........ The examination of the cultures made by inoculating the tubes with the exudation from the throat of .......... on ...... does not show the presence of any diphtheria bacilli.

The case is, therefore, not true diphtheria,[1] but pseudo or false diphtheria and no further cognizance will be taken of it by the department, unless by special request of the physician in attendance.

ERNEST WENDE, M. D., *Health Commissioner.*

............ *Bacteriologist.*

1. This conclusion is based on the supposition that the directions have been properly carried out and that the inoculation was made before the commencement of convalescence. After convalescence is established the bacilli often disappear from the exudate.

DEPARTMENT OF HEALTH,<br>BACTERIOLOGICAL LABORATORY.

BUFFALO, N. Y., ......... 189..

Dear Dr ........ The examination of the cultures made by inoculating the tube with the exudation from the throat of .... ..... on ...... does not admit of an exact bacteriological diagnosis, for the following reasons :

*A.* The inoculation was made at so late a period in the disease that it is possible that the diphtheria bacilli, though now absent, were at an earlier time present.

*B.* The growth on the culture media was so scanty that it is probable that the inoculation was not properly made, or that some antiseptic had been applied to the throat shortly before obtaining the material for inoculating the tube.

*C.* The culture media was badly contaminated.

*D.* The serum in the tube was too dry to permit of the growth of the diphtheria bacilli.

*a.* Another culture is requested.

*b.* The case will be treated as one of diphtheria.

*c.* The case will be treated as one of false diphtheria unless the physician in charge of the case requests otherwise.

ERNEST WENDE, M. D., *Health Commissioner.*

............ *Bacteriologist.*

NIAGARA WATER.

Besides the special work performed, pertaining to diphtheria, there is being made a daily examination of the Niagara tap water, with frequent examination of that coming directly from the reservoir, Niagara river and Lake Erie. It is the privilege of any physician or sanitarian residing in the city to submit such material to the department for bacteriological examination as may be thought detrimental to health.

TUBERCULOUS SPUTUM.

The examination of sputum for the physicians is being fully established and the following blank of instruction will accompany the circular pertaining to diphtheria.

DEPARTMENT OF HEALTH,
BACTERIOLOGICAL LABORATORY,
BUFFALO, N. Y.

*Directions for Collection of Sputum for Bacteriological Examination in Pulmonary Tuberculosis.*

Sputum should be collected only in clean, wide-mouthed, well-stoppered bottles, with a capacity of at least four ounces.

Care should be taken that bronchial and not pharyngeal secretion is collected, and the expectoration discharged early in the morning is to be preferred. If the expectoration is scanty the entire amount discharged in twenty-four hours should be collected.

Depending on the result obtained from the examination of sputum for tuberculosis, one of the following blanks is filled out and mailed to the physician who submitted the sample.

DEPARTMENT OF HEALTH, BACTERIOLOGICAL LABORATORY,
MUNICIPAL BUILDING, DELAWARE AVENUE,

BUFFALO, N. Y., ........189..

Dear Dr..........The examination of the sputum from.......... received on........shows the presence of the tubercle bacilli.

The case is, therefore, one of pulmonary tuberculosis.

ERNEST WENDE, *Health Commissioner.*

............ *Bacteriologist.*

DEPARTMENT OF HEALTH, BACTERIOLOGICAL LABORATORY.
MUNICIPAL BUILDING, DELAWARE AVENUE.

Buffalo, N. Y., .........189..

Dear Dr...........The examination of the sputum from..........
received on........does not show the presence of any tubercle bacilli.

It is not to be assumed, however, from the result of this examination, that the case is not one of possible pulmonary tuberculosis, if the family history and clinical symptoms indicate such disease, for frequently in this disease tubercle bacilli are at times absent from the sputum, and the disease can only be excluded if repeated examinations of sputum fail to show the presence of bacilli. If this case is still regarded as possibly tuberculosis, other specimens should be sent for examination.

It should be kept constantly in mind, that the demonstrations of the presence of tubercle bacilli in the sputum proves conclusively the existence of tuberculosis, but the absence of tubercle bacilli, or the failure to find them microscopically, does not exclude the disease.

ERNEST WENDE, M. D., *Health Commissioner.*

............. *Bacteriologist.*

REFERENCES.

1. Klebs—*Verhandl. des Zweiten Congress f. Inn. Medicin*, 1883.
2. Löffler—*Mitth. aus d. Kais. Gesundheitsamte, Bd.* 2, 1884.
3. Roux and Yersin—*Annales de L'Inst. Pasteur II.*, 1888, page 629.
4. Welch and Flexner—*Johns Hopkins Bulletin*, October, 1891.
5. Prudden—*Medical Record*, April 18, 1891.
6. Babes—*Virch. Archiv., Bd.* 119, *S.* 463.
7. *Scientific Bulletin No.* 1, Health Department of City of New York.

